# MicroRNA Alterations in a Tg501 Mouse Model of Prion Disease

**DOI:** 10.3390/biom10060908

**Published:** 2020-06-15

**Authors:** Janne M. Toivonen, David Sanz-Rubio, Óscar López-Pérez, Alba Marín-Moreno, Rosa Bolea, Rosario Osta, Juan J. Badiola, Pilar Zaragoza, Juan-Carlos Espinosa, Juan-Maria Torres, Inmaculada Martín-Burriel

**Affiliations:** 1 Laboratorio de Genética Bioquímica (LAGENBIO), Facultad de Veterinaria, University of Zaragoza, 50013 Zaragoza, Spain; davidsanzrubio91@gmail.com (D.S.-R.); oscarlzpz@gmail.com (Ó.L.-P.); osta@unizar.es (R.O.); pilarzar@unizar.es (P.Z.); minma@unizar.es (I.M.-B.); 2 Instituto de Investigación Sanitaria Aragón (IIS Aragón), University of Zaragoza, 50009 Zaragoza, Spain; 3 Instituto Agroalimentario de Aragón (IA2) University of Zaragoza-CITA, 50013 Zaragoza, Spain; 4 The Network Center for Biomedical Research in Neurodegenerative Diseases (CIBERNED), Institute Carlos III, 28031 Madrid, Spain; 5 Centro de Encefalopatías y Enfermedades Transmisibles Emergentes, University of Zaragoza, 50013 Zaragoza, Spain; rbolea@unizar.es (R.B.); badiola@unizar.es (J.J.B.); 6 Instituto de Investigación Biomédica de Bellvitge (IDIBELL), 08908 Barcelona, Spain; 7 Centro de Investigación en Sanidad Animal (CISA-INIA), 28130 Madrid, Spain; marin.alba@inia.es (A.M.-M.); espinosa.juan@inia.es (J.-C.E.); jmtorres@inia.es (J.-M.T.)

**Keywords:** prion diseases, scrapie, microRNA, biomarkers

## Abstract

MicroRNAs (miRNAs) may contribute to the development and pathology of many neurodegenerative diseases, including prion diseases. They are also promising biomarker candidates due to their stability in body fluids. We investigated miRNA alterations in a Tg501 mouse model of prion diseases that expresses a transgene encoding the goat prion protein (*PRNP*). Tg501 mice intracranially inoculated with mouse-adapted goat scrapie were compared with age-matched, mock inoculated controls in preclinical and clinical stages. Small RNA sequencing from the cervical spinal cord indicated that miR-223-3p, miR-151-3p, and miR-144-5p were dysregulated in scrapie-inoculated animals before the onset of symptoms. In clinical-stage animals, 23 significant miRNA alterations were found. These miRNAs were predicted to modify the Kyoto Encyclopedia of Genes and Genomes (KEGG) pathways including prion disease, extracellular matrix interactions, glutaminergic synapse, axon guidance, and transforming growth factor-beta signaling. MicroRNAs miR-146a-5p (up in cervical spinal cord) and miR-342-3p (down in cervical spinal cord, cerebellum and plasma), both indicated in neurodegenerative diseases earlier, were verified by quantitative real-time polymerase chain reaction (qRT-PCR). Minimal changes observed before the disease onset suggests that most miRNA alterations observed here are driven by advanced prion-associated pathology, possibly limiting their use as diagnostic markers. However, the results encourage further mechanistic studies on miRNA-regulated pathways involved in these neurodegenerative conditions.

## 1. Introduction

MicroRNAs (miRNAs) are short noncoding RNAs that regulate posttranscriptional gene expression by imperfect base pairing with target messenger RNAs (mRNAs), in most cases at the 3′ untranslated region. In metazoans, the major determinant for miRNA binding to these targets is a complementary seed sequence, a 6–8 nucleotide domain at the 5′ extremity of the miRNA. This miRNA-mRNA interaction typically causes translational repression and/or degradation of the target mRNA, subsequently manifesting as lowered levels of encoded protein. As translation from more than half of protein-coding genes in animals is regulated by miRNAs [1], it is not surprising that perturbations in miRNA expression or function are frequently associated with neurodegenerative diseases (NDD). These include transmissible spongiform encephalopathies (TSE, or prion diseases) [2], Alzheimer’s disease (AD), Parkinson’s disease, Huntington’s disease and amyotrophic lateral sclerosis (ALS) [3], all associated with accumulation and spreading of self-templating aggregates. Although the knowledge of miRNA alterations in these NDD has substantially advanced in the last ten years, the understanding of their role in these pathologies is still rudimentary, especially because for the great majority of the reported alterations it remains unclear whether or not they are functionally associated with etiopathogenesis of these diseases. However, some miRNAs up-regulated sporadic Creutzfeldt-Jakob disease (sCJD) brain have also been shown to be enriched in RNA induced silencing complexes (RISC), the essential machineries of miRNA function in vivo [4]. This suggests for the first time that the observed miRNA alterations are not merely bystanders but may bear relevance in prion disease-associated posttranslational perturbations.

Whether or not the influence of a certain miRNA on pathological process is known in molecular mechanistic angle, the occurrence of miRNA alterations in various disorders has created substantial expectations for their use as diagnostic and/or prognostic biomarkers. The relatively straightforward detection methods combined with the exceptional stability of miRNAs in the biofluids, such as blood plasma, cerebrospinal fluid (CSF), urine, or saliva, would ideally permit easily obtained diagnostic and/or prognostic tools for NDD. In the context of prion diseases such circulating biomarker candidates have been previously characterized in two targeted candidate studies: one in plasma of sheep naturally infected with scrapie [5] and another in CSF of sCJD patients [4]. Curiously, in both studies, the alterations observed in the biofluids were not observed in the central nervous system (CNS), suggesting the possibility that the tissue source(s) of altered miRNAs in biofluids are not necessarily those primarily involved in the pathogenesis. Recently, the first unbiased next-generation sequencing (NGS) -based study in biofluids indicated several miRNA alterations in serum of elk suffering from natural chronic wasting disease (CWD) and some of these were shown to be conserved in scrapie-infected hamsters [6]. This indicates that experimentally infected laboratory models, such as hamsters or mice, may bear relevance to the naturally affected animals and human prion diseases. As miRNA alterations are thought to be dynamic depending on the stage of the disease [7], a large variability in disease incubation time in natural prion infection may mask some differences that could be potentially observed in laboratory models.

Here, we have used Tg501 mouse model of scrapie to investigate preclinical and clinical-stage miRNA alterations in the CNS and blood plasma by NGS of small RNAs and quantitative real-time PCR (qRT-PCR). The results indicate that whereas preclinical alterations in vivo are few, some clinical-stage miRNA alterations frequently reported in NDD are present in the used model and could serve as targets for subsequent mechanistic studies and as biomarker candidates for natural human and animal prion disease. The cellular processes predicted to be affected by the observed miRNA alterations provide candidate pathways that could be involved in prion-induced pathology. 

## 2. Materials and Methods

### 2.1. Animals

Tg501 mice used for small RNA sequencing express wild-type goat *PRNP* allele under the control of murine PrP promoter [8] and were intracerebrally inoculated with Tg501-adapted caprine scrapie strain (Goat-Sc S2) at 50 days of age. Age-matched Tg501 animals inoculated with mock Tg501 CNS material were used as controls. Tg338 mice, which were used for qRT-PCR verification only, express a transgenic valine/arginine/glutamine (VRQ) allele of the ovine *PRNP* under the ovine PrP promoter [9,10] and were intracerebrally inoculated with Tg338-adapted classical scrapie CNS originally derived from ARQ/ARQ scrapie sheep or, alternatively, were mock inoculated with non-inoculated Tg338 CNS material, at approximately 50 days of age, as described earlier [11]. All experimental procedures were carried out in compliance with the recommendations for the care and use of experimental animals established by Spanish law (R.D. 53/2013) and European Directive 2010/63/UE. Experiments on Tg501 mice were approved by Ethics Committee for Animal Experimentation of the Spanish Instituto Nacional de Investigación y Tecnología Agraria y Alimentaria (INIA) (approval code PROEX263/15) and those performed in Tg338 animals were approved by the Ethics Committee for Animal Experimentation of the University of Zaragoza (approval code PI40/15). 

### 2.2. Tissue Collection

In all cases, half of the mice were sacrificed in the preclinical (non-symptomatic) stage and another half in the clinical (symptomatic) stage for tissue collection. In the case of Tg501, the mice were sacrificed, and the tissues were dissected at 100 days post-inoculation (DPI, preclinical stage) and 220 DPI (clinical stage) for both scrapie-inoculated and non-inoculated control groups. The tissues from Tg338 model were collected as described earlier [11]. After euthanasia, blood was extracted to EDTA-containing tubes by cardiac puncture and centrifuged at 3000 rpm for 10 min, after which the plasma fraction was collected to nuclease-free eppendorf tubes, frozen in dry ice and stored at −80 °C. Cervical spinal cord and cerebellum were dissected, preserved in RNAlater (Thermo Fisher Scientific, Waltham, MA, USA), kept 24 h in +4 °C and then stored at −80 °C. The collected Tg501 cervical spinal cord tissue was used for small RNA sequencing and qRT-PCR verification, whereas the cerebellum and blood plasma for qRT-PCR studies only.

### 2.3. RNA Extraction

RNAlater—stored solid tissue was dried from excess RNAlater solution with Whatman filter paper, submerged in 0.5 mL Qiazol (Qiagen, Hilden, Germany) in TeSeE grinding tubes (Bio-Rad, Hercules, CA, USA) and homogenized using TeSeE PRECESS 48 Homogenizer (Bio-Rad). The total RNA from the homogenate was purified by Direct-zol RNA Miniprep column purification (Zymo Research, Irvine, CA, USA) according to the providers’ protocol. The RNA was eluted in 50 µL of nuclease-free water and stored in −80 °C for further use. Circulating RNA from plasma samples was purified using Norgen Total RNA kit (Norgen Biotek, Thorold, ON, Canada) according to the modified protocol for plasma samples by the manufacturer. Briefly, thawed plasma samples were centrifuged at 400× *g* for 2 min and 100 µL of cell-free plasma was transferred to a new nuclease-free eppendorf tube. After the denaturation step, 25 fmol synthetic *C. elegans* miRNA cel-miR-39-3p (Qiagen) was spiked in for normalization together with 0.7 µL MS2 RNA carrier (Roche). Instead of using the provided washing solutions, washing steps were done using 95% ethanol and the RNA was eluted and stored as above. 

### 2.4. miRNA Sequencing and Data Analysis

The small RNA sequencing analysis was performed on a total of 24 cervical spinal cord samples from Tg501 mice. This consisted of 7 replicates for clinical control, 5 replicates for clinical inoculated and 6 replicates for preclinical control and for preclinical inoculated. The RNA quality control (Figure S1) was performed using NanoDrop (Thermo Fischer) and 2100 Bioanalyzer (Agilent, Santa Clara, CA, USA). Sequencing libraries were prepared using TruSeq Small RNA Library Preparation Kit (Illumina, San Diego, CA, USA) and sequenced as 50bp single-end reads with HiSeq v4 chemistry on Illumina HiSeq 2500 high-throughput sequencing system and finally inspected for quality using FastQC v0.11.2. Raw, compressed fastq.gz files were cleaned from 3′ sequencing adapters, aligned with miRBase21 [12] and counted with Oasis 2.0 sRNA detection module [13,14] and Chimira [15], both using DESeq2 algorithm [16] to produce small RNA count files. The mature miRNA count was carried out using the two platforms because i) Oasis 2.0 allows the downstream differential expression analysis and prediction of novel miRNAs and ii) Chimira can be used to detect potential miRNA modifications in the reads. As essentially identical read counts were produced by the two platforms (Table S1), Oasis 2.0 was used for the subsequent steps. Within Oasis 2.0, the reads between 15–19 nucleotides of length were aligned with 0 mismatches allowed and those of length 20–32 nucleotides were mapped allowing for 1 mismatch. Unmapped reads from the previous step were then aligned to the reference genome to predict novel miRNAs using miRDeep2 [17]. Principal component analysis from raw reads were carried out using Clustvis [18]. The differential expression analysis was carried out for each experimental stage from count files with Oasis 2.0 SmallRNA DE analysis module using min-max read length of 15–32 and base mean cutoff 50 for minimum number of reads (mean of all samples, separated in clinical and preclinical groups). Kyoto Encyclopedia of Genes and Genomes (KEGG) analysis was carried out for down-regulated and up-regulated miRNAs using DIANA miRPath v3.0 tool [19] using the microT-CDS option. The unmodified fastq.gz files and the count files for the raw reads are available for download in the Gene Expression Omnibus (GEO) series GSE146013.

### 2.5. Quantitative PCR

**Figure A1:**
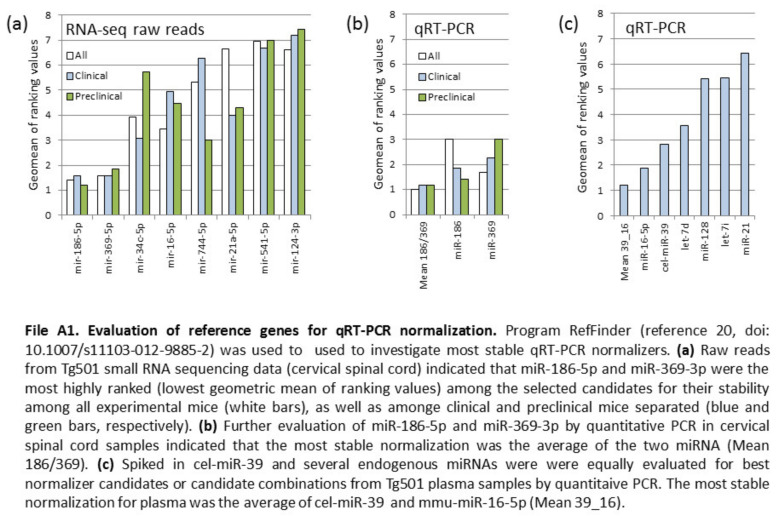
Evaluation of reference genes for qRT-PCR normalization.

To verify some of the results obtained from the RNA sequencing, qRT-PCR was carried out as previously described [5]. Briefly, miRNA cDNA was prepared from each sample with 30 ng of template RNA using TaqMan MicroRNA Reverse Transcription Kit (Thermo Fisher Scientific). For plasma samples, the same volume of spiked-in plasma RNA was directly used as a template. Gene expression was measured using standard fast conditions in StepOne Plus Real-Time PCR instrument (Applied Biosystems, Foster City, CA, USA) in triplicate reactions consisting of 2.25 µL of 1:7-diluted cDNA, 0.25 µL of TaqMan MicroRNA Assay (listed in Table S2), 2.5 µL of TaqMan Fast Universal PCR Master Mix (2X), no AmpErase™ UNG (Thermo Fisher Scientific). The CNS tissue samples were normalized with the mean of miR-369-5p and miR-186-5p and the plasma samples with the mean of miR-16-5p and spiked-in cel-miR-39-3p as these combinations showed the greatest stability among the samples when several candidates were tested by RefFinder (see also Appendix A, Figure A1) [20]. Relative expression between the prion-inoculated and the control mice was calculated as previously described [5].

## 3. Results

### 3.1. Small RNA Sequencing 

RNA extractions from cervical spinal cord tissue of Tg501 mice resulted in good quality RNA as shown by absorbance ratios and RNA integrity values (Figure S1). Small RNA sequencing resulted in an average of approximately ten million reads per sample (range 6.9 × 10^6^–1.4 × 10^7^ reads) with an error probability of ≤ 0.001 in more than 97% of the base calls (Phred Q-score ≥ 30). The numerical data on mature miRNA detection carried by two platforms, Chimira and Oasis 2.0, are summarized in Table S1. From the detected mature miRNAs, 46% were present in extremely low levels with average of 1–10 reads per sample (Figure S2a). As expected, those miRNAs representing moderate, high, and extremely high expression levels were present in progressively lower numbers. However, eight most highly expressed miRNA represented 49% of the total reads (Figure S2b). No significant alterations in miRNA edition or modifications were detected and alignment of reads that did not match to those annotated in miRBase to mouse genome (version GRMm38) did not reveal any novel miRNAs. The contribution of the other small RNA species (non-miRNAs) to the total read count was ~3%. 

**Figure 1:**
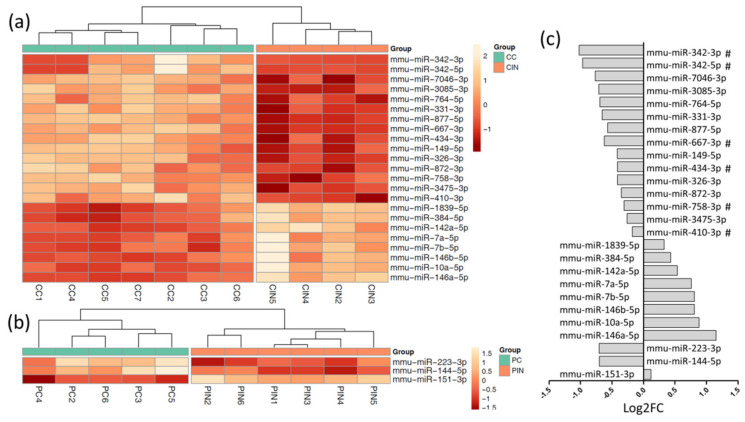
Differentially expressed (DE) miRNAs in Tg501 mice. (**a**) Heatmap of significant DE miRNAs (Benjamini–Hochberg padj < 0.05) in clinical-stage Tg501 mice compared with age-matched controls. (**b**) Significant DE miRNAs (padj < 0.05) in preclinical Tg501 mice compared with age-matched controls. (**c**) Log2 fold change of clinical and preclinical miRNA alterations. Down-regulated miRNAs clustered in chromosome 12 are indicated with hash (#). Abbreviations: CC, clinical control; CIN, clinical inoculated; PC, preclinical control; PIN, preclinical inoculated; Log2 FC, log2 fold change.

Principal component analysis identified two experimental animals that were classified as outliers (Figure S3) and were excluded from subsequent steps. From the total annotated mature miRNAs, 67 miRNAs in clinical samples and 32 miRNAs in preclinical samples displayed p-value < 0.05 between inoculated and their age-matched control groups. As shown in Figure 1, after multiple correction (Benjamini–Hochberg, adjusted *p* < 0.05), 23 miRNAs remained significant at clinical stage (Figure 1a) whereas only 3 miRNAs passed this in preclinical mice (Figure 1b). There was no overlap between the dysregulated miRNAs in the preclinical and clinical mice. In clinical stage, 8 miRNAs (miR-142a-5p, -7b-5p, -10a-5p, -7a-5p, -146b-5p, -146a-5p, -1839-5p and -384-5p) were up-regulated and 15 miRNAs (miR-342-3p, -342-5p, -764-5p, -410-3p, -758-3p, -3085-3p, -872-3p, -667-3p, -331-3p, -3475-3p, -7046-3p, -434-3p, -326-3p, and -877-5p) down-regulated. In preclinical mice, miR-223-3p and -144-5p out of three were down-regulated and miR-151-3p up-regulated (Figure 1c). All except four of these miRNAs are classified as high confidence miRNAs in mice and are listed in Table 1. No significant differences between experimental groups were found in other small RNA species.

Over-representation analysis by miRNA Enrichment Analysis and Annotation tool miEAA 2.0 [21] indicated that miRNAs encoded by chromosome 12 were enriched in those down-regulated by prion inoculation (*p* = 0.001, padj = 0.008). Indeed, 40% (6/15) of the significantly down-regulated miRNAs in clinical prion-inoculated mice were found to be encoded by chromosome 12 (labeled with # in Figure 1c). These included mature products of miR-342 (miR-342-3p, -342-5p) and four others (miR-667-3p, -434-3p, -758-3p, -410-3p) located in within activity induced *Dlk1-Dio3* locus of chromosome 12. The fact that all these miRNAs were down-regulated suggests that the spanning region could be co-regulated. Six additional miRNAs encoded by the same locus (miR-376a-5p, -377-3p, -666-5p, -666-3p, -496a-3p and -345-5p) also showed tendency for down-regulation (*p* < 0.05) but did not remain significant after multiple correction. 

### 3.2. Functional Annotation of Differentially Expressed miRNAs 

**Figure A2:**
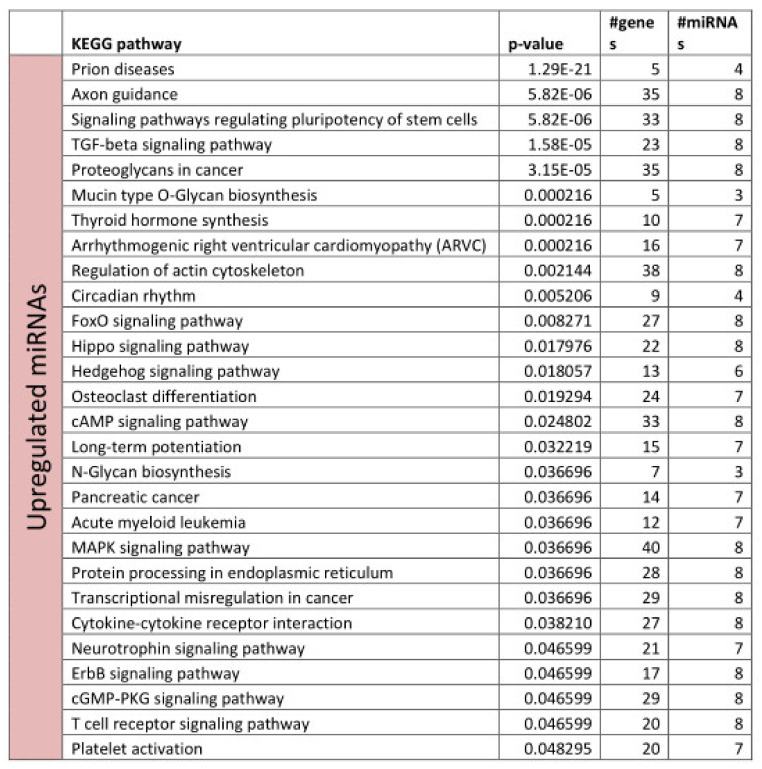

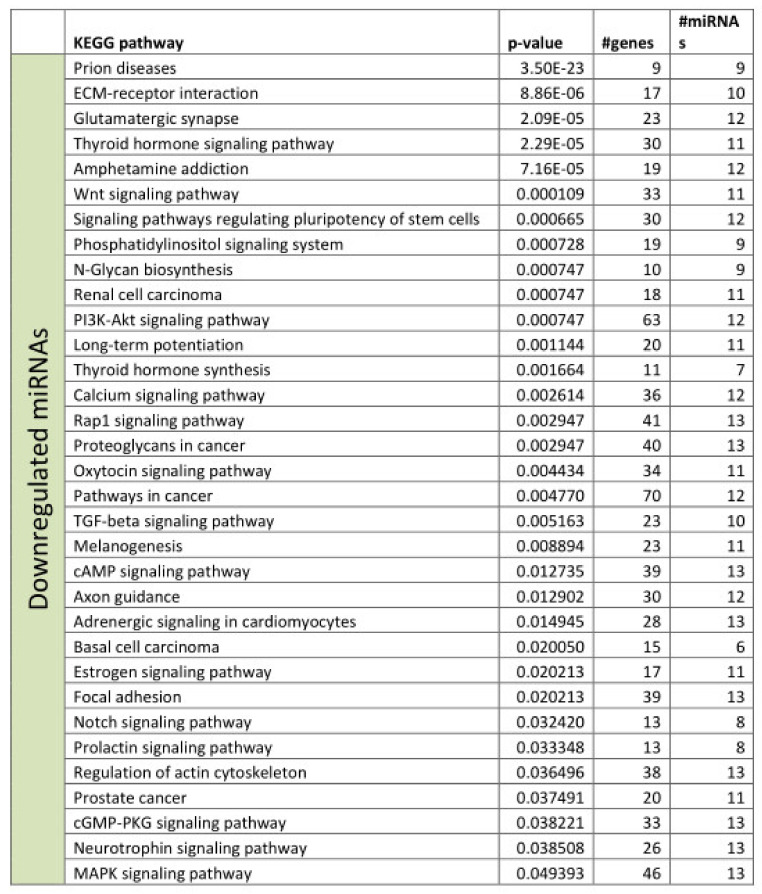

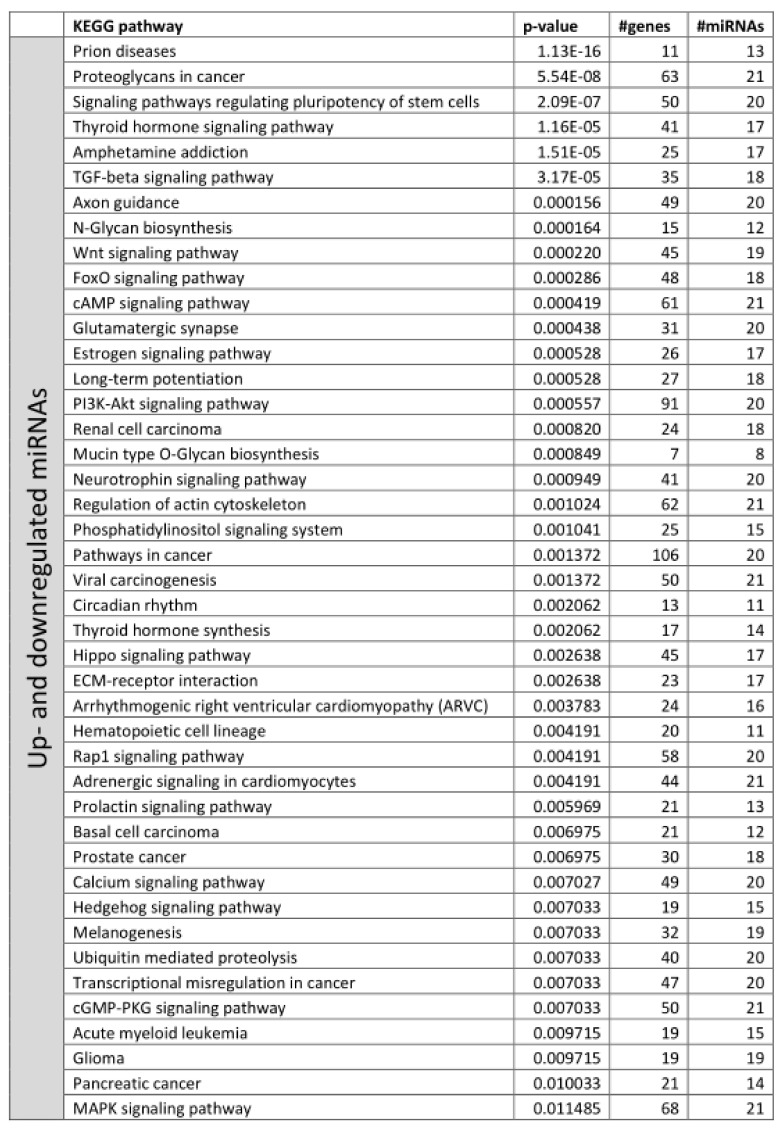

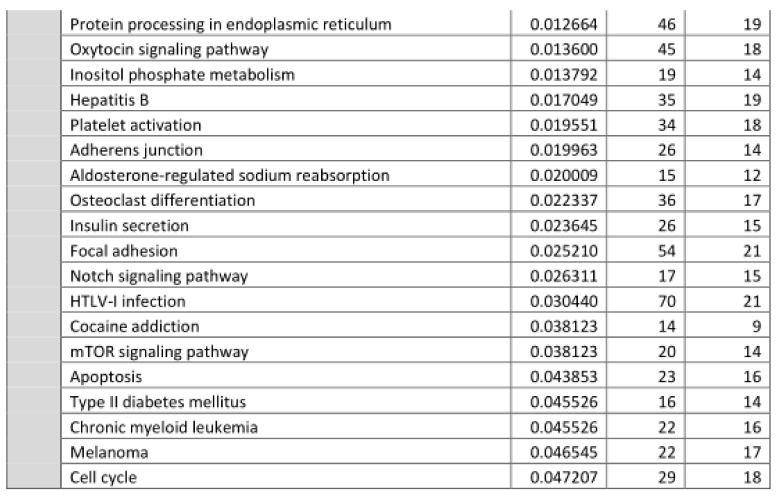
Full lists of the significantly enriched pathways from the functional annotation analysis of up-regulated, down-regulated, and combined DE miRNA data.

To investigate biological processes that could be affected based on predicted target genes of the altered miRNAs, KEGG analysis was carried out using DIANA miRPath v3.0 tool [19]. In the case of miRNAs, the expected consequence of a decreased miRNA abundance is the up-regulation of the target pathways, whereas reverse is true for the significant pathways associated with miRNAs that are increased. Therefore, the analysis was performed for up-regulated (Table 2) and down-regulated (Table 3) miRNAs separately. Curiously, in both cases the top KEGG pathway was prion diseases, a category which in mice contains 34 genes. Other shared pathways for the increased and decreased miRNAs included signaling pathways regulating pluripotency of stem cells and various pathways involved in protein glycosylation. Highly significant pathways of interest involving down-regulated miRNAs included extracellular matrix (ECM) interaction and glutamatergic synapse, whereas axon guidance and transforming growth factor-beta (TGF-beta) signaling were significant specifically in up-regulated miRNA group. A complete list of the significantly enriched pathways is presented in Appendix A, Figure A2.

### 3.3. Verification of miRNA Expression by QRT-PCR and Detection of Candidate Biomarkers from Plasma 

**Figure 2:**
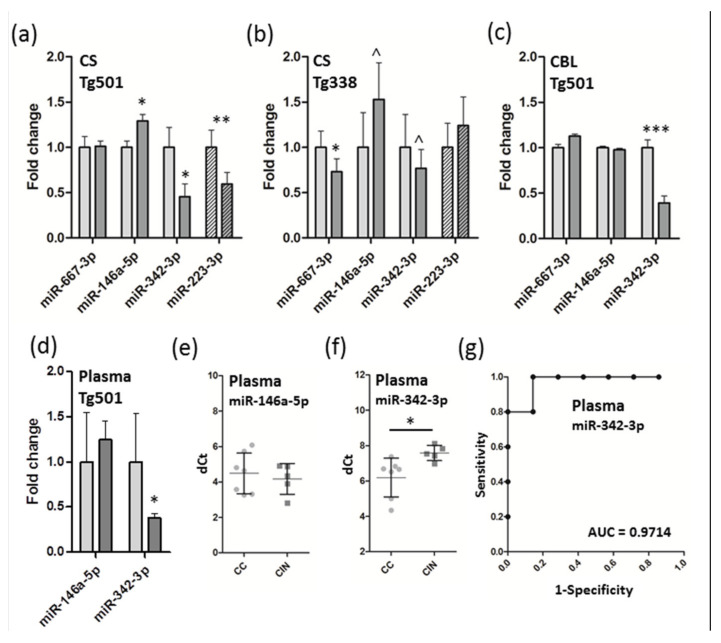
Quantitative PCR of differentially expressed miRNAs in Tg501 and Tg338 mice. Controls and inoculated groups are denoted with light grey and dark grey bars, respectively, whereas solid bars represent the clinical stage and striped bars the preclinical stage. (**a**) Fold change expression (+/- SD) of selected miRNAs in the cervical spinal cord of Tg501 mice, (**b**) cervical spinal cord of Tg338 mice, (**c**) cerebellum of Tg501 mice and (**d**) blood plasma of Tg501 mice. Plasma delta Ct (dCt) values for individual mice are indicated for (**e**) miR-146a-5p and (**f**) miR-342-3p. (**g**) Receiver operating characteristic (ROC) curve for plasma miR-342-3p. The CNS tissues were normalized to the mean of miR-186-5p and miR-369-5p, and that of plasma samples to the mean of miR-16-5p and synthetic Cel-miR-39 (spike-in). Student’s t-test, *p < 0.05, **p < 0.01, ***p < 0.001, ^*p* < 0.1 (tendency). Abbreviations: CS, cervical spinal cord; CBL, cerebellum; dCt, delta Ct; AUC, area under curve.

From the altered miRNAs, a subset was verified by qRT-PCR. Notably, mature miRNA miR-146a-5p increased (*p* < 0.05) and miR-342-3p decreased (*p* < 0.05) in cervical spinal cord of clinical prion-inoculated mice, and miR-223-3p decreased (*p* < 0.01) in preclinical inoculated mice (Figure 2a). However, miR-667-3p that was most significantly increased in the sequencing data could not be verified in Tg501 mice, although it was significantly down-regulated in another mouse model used, Tg338 (Figure 2b), where close to significant tendency was observed also for up-regulation of miR-146a-5p and down-regulation of miR-342-3p (both *p* < 0.1). However, miR-223-3p was not affected in the preclinical stage in this model. Finally, the levels of miR-342-3p but not those of miR-146a-5p decreased in the cerebellum (Figure 2c) and blood plasma (Figure 2d) of Tg501 mice. Although variation in individual experimental mice in plasma samples was relatively high in both miR-146a-5p (Figure 2e) and miR-342-3p (Figure 2f), the receiver operating characteristic (ROC) analysis revealed that the levels of plasma miR-342-3p correctly identified prion-infected Tg501 mice in the majority samples.

## 4. Discussion

The presented data demonstrates that in the Tg501 mouse model of prion disease, the miRNA alterations caused by inoculation of scrapie prion are mainly observed in clinical-stage animals with few significant alterations in the preclinical stage. This indicates that in the used model, most observed changes are likely related to the late pathological alterations in the CNS, as also observed recently in the Tg340 model of sCJD [4]. Although this may possibly limit the use of such models in the search for early diagnostic miRNA markers, it should be noted that all miRNAs altered in preclinical Tg501 mice were also dysregulated in predominantly asymptomatic CWD elk [6]. Additionally, clinically altered miRNAs found to be conserved markers of prion infection could be useful for disease confirmation together with other diagnostic tests. Unbiased circulating miRNA experiments, i.e., those not relying on pre-determined miRNA alterations in the CNS, should be encouraged to reveal the whole spectrum of potential preclinical biomarkers present in Tg501 and similar mouse models

Most mammalian miRNA genes are scattered across the genome, but some are organized in clusters. Several miRNAs down-regulated in the clinically affected prion-inoculated mice were found to be encoded by chromosome 12, containing the largest mammalian miRNA mega-cluster [22]. This ~850 kb *Dlk-Dio3* imprinted region encompasses three long-noncoding RNAs and 54 miRNAs whose mis-expression miRNAs may disturb neuronal homeostasis and/or neurogenesis and this way contribute to the pathogenesis of neurodevelopmental and neuropsychiatric disorders [23]. Several *Dlk-Dio3* –encoded miRNAs have been also shown to be down-regulated in aging skeletal muscle and related to the accompanying muscle decline [24,25]. Therefore, further studies on potential mechanistic basis and involvement of this region and derived miRNAs on prion diseases and other NDD are warranted. 

Roughly half of the significantly altered miRNAs observed here have been mentioned in the context of prion diseases earlier (Table 4). Most notably, these include miR-342-3p and miR-146a-5p that belong to the most commonly found alterations in the prion diseases and related neurodegenerative conditions. miR-342-3p was first shown to be up-regulated in post-mortem biopsies of the bovine spongiform encephalopathy (BSE)-infected macaque and sCJD patients [26]. Although limited for the number of biological replicates, these studies gave first indications that some miRNAs may be commonly regulated in experimentally induced and idiopathic prion diseases. Clinical-stage increase of miR-342-3p has been since documented in prion strain 22A-inoculated mouse brain [27], various brain areas of sCJD MM1 mouse model [4], plasma from naturally infected scrapie sheep [5] and exosomes from prion-infected neurons in culture [28]. MicroRNA miR-342-3p is also up-regulated in the brain of transgenic models of AD [29], where its inhibition in hippocampus reduces β-amyloid plaques and ameliorates learning and memory [30]. Here, miR-342-3p was found down-regulated in cervical spinal cord, cerebellum, and blood plasma of clinical prion-inoculated Tg501 mice, which in contrast with most previous studies on prion disease and its models. However, miR-342-3p is decreased in total serum [31] and serum and plasma-derived exosomes [32,33] of AD patients where its levels correlate with disease duration [31]. Similarly, miR-342-3p is down-regulated in peripheral blood of sporadic ALS patients [34]. This indicates that although miR-342-3p alterations are commonly observed in NDD and their models, the direction of the regulation may be tissue-specific or temporally affected by the disease course. 

MicroRNA miR-146a-5p is generally found up-regulated in experimentally induced prion disease in mice and human TSE patients (Table 4) [4,7,27,35,36,37,38]. Its up-regulation seems to be, in most cases, exclusive to the clinical stages, as shown recently in sCJD MM1 mouse model [4]. As an exception, miR-146-5p was down-regulated in prion-infected cultured neuronal cells and their secreted exosomes [28] which could possibly reflect in vitro conditions. However, it should be considered that TSE-associated miRNA alterations may manifest in specific spatiotemporal sequence. Indeed, dynamic regulation of miR-146-5p seems to occur depending on the disease stage as both increase and decrease have been documented in the hippocampal CA1 neurons [7] and forebrain synaptoneurosomes [35] of prion-infected mice. A subset of up-regulated miRNAs in the clinical prion-inoculated Tg501 mice are also increased in AD patients and mouse models of AD. The miRNAs miR-146a-5p, -142a-5p and -10a-5p show common increase in the hippocampi of transgenic APP (APPswe/PS1L166P) and TAU (THY-Tau22) mice, and the levels of miR-146a-5p and -142a-5p are also elevated in the post-mortem hippocampal tissue of AD patients [39]. The same study also found consistent increase in the two mouse models and the AD samples in the levels of miR-155-5p which in Tg501 mice showed a tendency for up-regulation (*p* = 0.003, padj = 0.06). miR-146a-5p and miR-155-5p are frequently cited as proinflammatory miRNAs and is up-regulated in temporal neocortex of AD patients [40] and in the spinal cord of sporadic and familial human ALS patients [41]. As observed in Tg501 mice, a recent study concluded that plasma levels of miR-146a-5p are not altered in AD patients [42] although contradictory results also exist [43,44].

From the diagnostic point of view, the most important miRNA alterations are those observed in the preclinical animals as they may have a role in the etiopathogenesis of the prion diseases and could possibly serve as early disease biomarkers. In the preclinical Tg501 mice, miR-223-3p was found to be significantly decreased in the cervical spinal cord. This miRNA has been previously seen affected in blood and blood-derived components of several NDD, including AD, ALS and both natural and experimental demyelinating diseases [46]. For example, miR-223 has been previously suggested as a possible biomarker in AD as it is down-regulated in serum and CSF of AD patients [47,48]. miR-223-3p is also well documented posttranscriptional regulator of NLRP3 inflammasome, a multiprotein signaling platforms sensing microbe- or damage-associated molecular patterns [49]. Although inflammasome activation seems to promote neurodegenerative conditions [50], it is not currently known miR-223-NLRP3 regulatory axis is present in the CNS tissue. Although nothing is known about the potential role of miR-151 in NDD, miR-144 has been shown to increase environmental stress susceptibility and is considered a risk factor for development of NDD such as AD [51]. Despite the low number of preclinical Tg501 miRNA alterations found, miR-223-3p, -151-3p and -144-5p were all also altered in serum from predominantly asymptomatic CWD elk population (Table 4) [6]. This provides some support for the consistency of the findings of presymptomatic miRNA alterations between TSE models and naturally infected animals. 

The most significantly enriched KEGG pathway for both up-regulated miRNAs (pathway expected to be repressed) and down-regulated miRNAs (pathway expected to be stimulated) was prion diseases. One third of the total genes in this pathway were identified as putative targets for the dysregulated miRNAs. The up/down miRNA datasets overlapped with regard to subset of target mRNAs that included genes involved in endoplasmic reticulum stress (Casp12) [52], modulation of astrocyte activation in murine scrapie model (Il1) [53], as well as cellular prion protein (PrPC)-associated lipid raft component (Fyn) [54] and Prnp itself. How the mixed up-/down-regulation of miRNAs targeting same transcripts translates to the target mRNA/protein levels needs further experimental validation. Additional genes that were predicted targets only to the down-regulated miRNAs (targets stimulated), included members of the complement membrane attack complex (C6 and C8g), a component of the innate immune system that correlates with neuropathological lesions in sCJD brains [55], a heat shock protein with protective role in prion disease (Hspa1a) [56], and a proinflammatory cytokine increased in patients with sCJD (Il6) [57]. 

High-scoring pathways in the group of down-regulated miRNAs (pathway stimulated) included “ECM-receptor interaction” and Glutaminergic synapse”. ECM compartment is thought to be critical for prion replication/infection as prominent components of ECM that localize with pathological PrP in the brain can inhibit prion propagation as well as enhance endocytosis of PrPC [58,59]. Genes implicated in the organization of the ECM are also affected in natural scrapie-infected sheep [60,61] and BSE [62]. Interactions between cells and the ECM are mediated by transmembrane molecules that regulate activities such as adhesion, migration, differentiation, proliferation, and apoptosis. Transmembrane integrins join the cells actin cytoskeleton to the ECM by binding adhesion molecules and structural proteins, including collagens, and couple this function with signal transduction activation. MicroRNA targets in this group included several integrins and collagens. Knockdown of integrin Itga8, a predicted target of miR-877-5p and -410-3p, is sufficient to convert prion-resistant neuroblastoma cells to a susceptible phenotype [63]. Of note, many transmembrane ECM proteins are also structural components of extracellular vesicles [64], secreted membrane-bound vehicles that exchange various biomolecules between cells, and PrPC expression has been shown to stimulate exosome secretion in some setups [65]. Glutaminergic synapse pathway has been linked to sCJD risk in a recent large genome-wide association study [45]. Specifically, protein metabotropic glutamate receptor mGluR8 (Grm8) associates with heightened Creutzfeldt-Jakob disease risk and was found here to be putative target genes for miR-342-3p. mGluR family is involved in the transduction of physiological and cytotoxic signals mediated by PrPC including neurite outgrowth and cellular toxicity of soluble β-amyloid oligomers [66,67]. 

High-scoring pathways in the group of up-regulated miRNAs (pathway repressed) included “Axon guidance” and “TGF-beta signaling”. Genes contributing to axon guidance are involved in a neural developmental process that helps axons extend to their correct targets and also play an important role in inflammation of the nervous system [68]. Down-regulation of axon guidance has been indicated as a late perturbation in three mouse models of prion disease [69] and in a case study of post-mortem brain from genetic CJD patient [70]. Here, the predicted targets included secreted proteins that guide the axonal tracts in the forebrain [71], as well as several ephrin receptors and semaphorins that represent molecular signals controlling multiple aspects of the regenerative response to CNS injury, and thus may also play a role protective role in prion-induced neural degeneration [68]. TGF-beta signaling pathway, potentially implicated in BSE [62], responds to structurally related secreted cytokines to modulate cellular functions (proliferation, apoptosis, differentiation and migration) through their plasma membrane receptors and result in activation of downstream signals leading to gene activation. One fourth of the total genes in this pathway were predicted targets and included several TGF-beta signaling receptors and downstream signal transducers. 

When the up- and down-regulated miRNA groups were combined and the analysis was repeated (see Appendix A, Figure A2), 11 pathways, including prion diseases, ECM-interactions, and TGF-beta signaling, were found common with total of 15 predicted pathways that were reported affected by miRNAs dysregulated in CWD [6]. This overlap is quite remarkable considering that none of the miRNAs included in the functional annotation analysis were the same in the two studies. Although direct comparisons are not possible because different tissues (CNS vs. circulating serum) were analyzed in the two studies, the observed overlap indicates that similar miRNA mediated processed may be affected in TSE and their models even though the specific miRNAs that regulate the pathways differ. 

## 5. Conclusions

Mouse model Tg501 of prion disease indicates several miRNAs, many of which are already described in the context of prion diseases and other NDD, altered in the clinical-stage mice, and very few alterations before the onset of symptoms. This may limit the use of the model for in the search for early presymptomatic biomarkers, although to definitively conclude this unbiased methodology should be used to directly measure miRNA alterations in easily accessible tissues such as blood, serum of plasma. However, the trend observed for co-regulation of several clustered miRNAs in Chromosome 12 warrants further investigation on its molecular basis, such as potential co-regulation by transcription factors or epigenetic modifications such as DNA methylation. Finally, functional studies on miRNA-mRNA networks implicated in the altered pathways on clinical mice are warranted to confirm the function of the predicted interactions and reveal the biological processes altered. 

## Figures and Tables

**Table 1:** Differentially expressed miRNAs in cervical spinal cord of Tg501 mice.

miRNA	Regulation/Stage	Log2 FC ^1^	*p*-value	padj ^2^	Hi-conf ^3^
mmu-miR-667-3p	down/clinical	−0.57	2.38 × 10^−10^	1.45 × 10^−7^	x
mmu-miR-146a-5p	up/clinical	1.15	7.46 × 10^−10^	2.28 × 10^−7^	x
mmu-miR-877-5p	down/clinical	−0.62	2.22 × 10^−9^	4.52 × 10^−7^	
mmu-miR-10a-5p	up/clinical	0.88	1.67 × 10^−8^	2.55 × 10^−6^	x
mmu-miR-142a-5p	up/clinical	0.54	4.81 × 10^−7^	5.87 × 10^−5^	
mmu-miR-3085-3p	down/clinical	−0.71	1.40 × 10^−6^	1.24 × 10^−4^	x
mmu-miR-331-3p	down/clinical	−0.66	1.42 × 10^−6^	1.24 × 10^−4^	
mmu-miR-7046-3p	down/clinical	−0.77	2.72 × 10^−6^	2.07 × 10^−4^	x
mmu-miR-1839-5p	up/clinical	0.33	2.06 × 10^−5^	1.40 × 10^−3^	x
mmu-miR-326-3p	down/clinical	−0.41	2.69 × 10^−5^	1.64 × 10^−3^	x
mmu-miR-434-3p	down/clinical	−0.41	7.06 × 10^−5^	3.92 × 10^−3^	x
mmu-miR-149-5p	down/clinical	−0.41	1.03 × 10^−5^	5.24 × 10^−3^	x
mmu-miR-7b-5p	up/clinical	0.81	1.26 × 10^−4^	5.78 × 10^−3^	x
mmu-miR-764-5p	down/clinical	−0.69	1.32 × 10^−4^	5.78 × 10^−3^	x
mmu-miR-146b-5p	up/clinical	0.81	1.85 × 10^−4^	7.56 × 10^−3^	x
mmu-miR-3475-3p	down/clinical	−0.26	2.07 × 10^−4^	7.90 × 10^−3^	
mmu-miR-758-3p	down/clinical	−0.31	2.79 × 10^−4^	1.00 × 10^−2^	x
mmu-miR-7a-5p	up/clinical	0.76	4.45 × 10^−4^	1.44 × 10^−2^	x
mmu-miR-384-5p	up/clinical	0.43	4.48 × 10^−4^	1.44 × 10^−2^	x
mmu-miR-410-3p	down/clinical	−0.18	7.15 × 10^−4^	2.18 × 10^−2^	x
mmu-miR-342-3p	down/clinical	−1.02	1.00 × 10^−3^	2.91 × 10^−2^	x
mmu-miR-872-3p	down/clinical	−0.35	1.11 × 10^−3^	3.07 × 10^−2^	x
mmu-miR-342-5p	down/clinical	−0.97	1.65 × 10^−3^	4.38 × 10^−2^	x
mmu-miR-223-3p	down/preclinical	−0.70	1.01 × 10^−4^	3.46 × 10^−2^	x
mmu-miR-151-3p	up/preclinical	0.12	1.40 × 10^−4^	3.46 × 10^−2^	x
mmu-miR-144-5p	down/preclinical	−0.70	1.70 × 10^−4^	3.46 × 10^−2^	x

^1^ Log2 fold change, ^2^ Adjusted p-value, ^3^ High-confidence miRNAs in miRBase.

**Table 2:** KEGG pathways for the up-regulated miRNAs in clinical-stage Tg501 mice (padj < 0.001).

Rank	KEGG pathway	padj	#genes	#miRNAs
1	Prion diseases	1.3 × 10^−21^	5	4
2	Axon guidance	5.8 × 10^−6^	35	8
3	Signaling regulating pluripotency of stem cells	5.8 × 10^−6^	33	8
4	TGF-beta signaling pathway	1.6 × 10^−5^	23	8
5	Proteoglycans in cancer	3.2 × 10^−5^	35	8
6	Mucin type O-Glycan biosynthesis	2.2 × 10^−4^	5	3
7	Thyroid hormone synthesis	2.2 × 10^−4^	10	7
8	Arrhythmogenic right ventricular cardiomyopathy	2.2 × 10^−4^	16	7

**Table 3:** KEGG pathways for the down-regulated miRNAs in clinical-stage Tg501 mice (padj < 0.001).

Rank	KEGG pathway	padj	#genes	#miRNAs
1	Prion diseases	3.5 × 10^−23^	9	9
2	ECM-receptor interaction	8.9 × 10^−6^	17	10
3	Glutamatergic synapse	2.1 × 10^−5^	23	12
4	Thyroid hormone signaling pathway	2.3 × 10^−5^	30	11
5	Amphetamine addiction	7.2 × 10^−5^	19	12
6	Wnt signaling pathway	1.1 × 10^−4^	33	11
7	Signaling regulating pluripotency of stem cells	6.7 × 10^−4^	30	12
8	Phosphatidylinositol signaling system	7.3 × 10^−4^	19	9
9	N-Glycan biosynthesis	7.5 × 10^−4^	10	9
10	Renal cell carcinoma	7.5 × 10^−4^	18	11
11	PI3K-Akt signaling pathway	7.5 × 10^−4^	63	12
12	Long-term potentiation	1.0 × 10^−3^	20	11

**Table 4:** Tg501miRNA alterations described earlier in the context of prion diseases.

Stage	miRNA	Tg501^1^	Pub^2^	Disease; Model(s)	Tissues or Fractions^3^	Refs.
Clinical Tg501	miR-667-3p	↓	↓	Prion (RML)—infected mice	FB synaptoneurosomes	[35]
	miR-146a-5p	↑	↑↓	sCJD; prion(RML/22A/ sCJD-MM1)—infected mice, prion(M1000)-infected neuronal cells	B, FC, CBL, FB synaptoneurosomes, neuronal cells, and exosomes	[4,7,27,28,35,38]
	miR-877-5p	↓	↓	sCJD; prion(sCJD-MM1)—infected mice	FC, CBL	[4]
	miR-142a-5p	↑	↑	Prion(RML)—infected mice	FB synaptoneurosomes	[35]
	miR-331-3p	↓	↓	sCJD; prion(sCJD-MM1)—infected mice	FC, C	[4]
	miR-149-5p	↓	↓	Prion(RML)—infected mice	FB, SN	[35]
	miR-7b-5p	↑	↑	Prion(RML)—infected mice	SN	[7,35]
	miR-146b-5p	↑	↓	Prion(RML)—infected mice	HC synaptoneurosomes	[35]
	miR-7a-5p	↑	↑	Prion(RML)-infected mice	FB synaptoneurosomes	[35]
	miR-342-3p	↓	↑↓	sCJD, sheep scrapie; prion(22A/sCJD-MM1)—infected mice, prion(BSE)—infected macaques, prion(M1000)—infected neuronal cells	FC, plasma; B,C, CBL, BP, neuronal cell-derived exosomes	[4,5,26,28,45]
	miR-342-5p	↓	↓	Prion (RML)—infected mice	HC synaptoneurosomes	[35]
Preclin. Tg501	miR-223-3p	↓	↑↓	Asymptomatic CWD elk; scrapie-infected hamster	Serum	[6]
	miR-151-3p	↑	↑	Asymptomatic CWD elk	Serum	[6]
	miR-144-5p	↓	↑	Asymptomatic CWD elk	Serum	[6]

^1^Arrow up indicates up-regulated and arrow down down-regulated miRNA in this study. ^2^As previously, but in earlier published studies. ^3^Abbreviations for the tissues: FB, forebrain; B, brain; FC, frontal cortex; cerebellum, CBL; C, cortex; HC, hippocampus; CSF, cerebrospinal fluid; BP, basis pontis.

## Supplementary Materials

The following are available online at https://www.mdpi.com/2218-273X/10/6/908/s1, Table S1: Comparison of miRNA detection carried by the two platforms, Table S2: List of miRNAs verified by qRT-PCR and corresponding TaqMan Assays used in the study, Figure S1: Quality control of cervical spinal cord RNA samples from Tg501 mice, Figure S2: Read count range and most abundant miRNAs in the cervical spinal cord samples, Figure S3: Principal component analysis of read counts from experimental mice.

## References

[1] Friedman R., Farh K.K.-H., Burge C.B., Bartel B. (2008). Most mammalian mRNAs are conserved targets of microRNAs. Genome Res..

[2] Kanata E., Thüne K., Xanthopoulos K., Ferrer I., Dafou D., Zerr I., Sklaviadis T., Llorens F. (2018). MicroRNA Alterations in the Brain and Body Fluids of Humans and Animal Prion Disease Models: Current Status and Perspectives. Front. Aging Neurosci..

[3] Maciotta S., Meregalli M., Torrente Y. (2013). The involvement of microRNAs in neurodegenerative diseases. Front. Cell. Neurosci..

[4] Llorens F., Thüne K., Marti E., Kanata E., Dafou D., Diaz-Lucena D., Vivancos A., Shomroni O., Zafar S., Schmitz M. (2018). Regional and subtype-dependent miRNA signatures in sporadic Creutzfeldt-Jakob disease are accompanied by alterations in miRNA silencing machinery and biogenesis. PLOS Pathog..

[5] Rubio D.S., López-Pérez Ó., Pablo, Álvaro D.A., Bolea R., Osta R., Badiola J.J., Zaragoza P., Martín-Burriel I., Toivonen J.M. (2017). Increased circulating microRNAs miR-342-3p and miR-21-5p in natural sheep prion disease. J. Gen. Virol..

[6] Slota J.A., Medina S.J., Klassen M., Gorski D., Mesa C.M., Robertson C., Mitchell G., Coulthart M.B., Pritzkow S., Soto C. (2019). Identification of circulating microRNA signatures as potential biomarkers in the serum of elk infected with chronic wasting disease. Sci. Rep..

[7] Majer A., Medina S.J., Niu Y., Abrenica B., Manguiat K.J., Frost K.L., Philipson C.S., Sorensen D.L., Booth S. (2012). Early Mechanisms of Pathobiology Are Revealed by Transcriptional Temporal Dynamics in Hippocampal CA1 Neurons of Prion Infected Mice. PLOS Pathog..

[8] Aguilar-Calvo P., Espinosa J.C., Pintado B., Gutiérrez-Adán A., Alamillo E., Miranda A., Prieto I., Bossers A., Andreoletti O., Torres J.M. (2014). Role of the goat K222-PrP(C) polymorphic variant in prion infection resistance. J. Virol..

[9] Laude H., Vilette D., Le Dur A., Archer F., Soulier S., Besnard N., Essalmani R., Vilotte J.-L. (2002). New in vivo and ex vivo models for the experimental study of sheep scrapie: Development and perspectives. Comptes Rendus Boil..

[10] Vilotte J.-L., Soulier S., Essalmani R., Stinnakre M.-G., Vaiman D., Lepourry L., Da Silva J.C., Besnard N., Dawson M., Buschmann A. (2001). Markedly Increased Susceptibility to Natural Sheep Scrapie of Transgenic Mice Expressing Ovine PrP. J. Virol..

[11] López-Pérez Ó., Toivonen J.M., Otero A., Solanas L., Zaragoza P., Badiola J.J., Osta R., Bolea R., Martín-Burriel I. (2019). Impairment of autophagy in scrapie-infected transgenic mice at the clinical stage. Lab. Investig..

[12] Kozomara A., Birgaoanu M., Griffiths-Jones S. (2018). miRBase: From microRNA sequences to function. Nucleic Acids Res..

[13] Capece V., Vizcaino J.C.G., Vidal R., Rahman R.-U., Centeno T.P., Shomroni O., Suberviola I., Fischer A., Bonn S. (2015). Oasis: Online analysis of small RNA deep sequencing data. Bioinform..

[14] Rahman R.-U., Gautam A., Bethune J., Sattar A., Fiosins M., Magruder D.S., Capece V., Shomroni O., Bonn S. (2018). Oasis 2: Improved online analysis of small RNA-seq data. BMC Bioinform..

[15] Vitsios D.M., Enright A.J. (2015). Chimira: Analysis of small RNA sequencing data and microRNA modifications. Bioinform..

[16] Love M.I., Huber W., Anders S. (2014). Moderated estimation of fold change and dispersion for RNA-seq data with DESeq2. Genome Biol.

[17] Friedländer M.R., Mackowiak S., Li N., Chen W., Rajewsky N. (2011). miRDeep2 accurately identifies known and hundreds of novel microRNA genes in seven animal clades. Nucleic Acids Res..

[18] Metsalu T., Vilo J. (2015). ClustVis: A web tool for visualizing clustering of multivariate data using Principal Component Analysis and heatmap. Nucleic Acids Res..

[19] Vlachos I.S., Zagganas K., Paraskevopoulou M.D., Georgakilas G., Karagkouni D., Vergoulis T., Dalamagas T., Hatzigeorgiou A.G. (2015). DIANA-miRPath v3.0: Deciphering microRNA function with experimental support. Nucleic Acids Res..

[20] Xie F., Xiao P., Chen N., Xu L., Zhang B. (2012). miRDeepFinder: A miRNA analysis tool for deep sequencing of plant small RNAs. Plant. Mol. Boil..

[21] Backes C., Khaleeq Q.T., Meese E., Keller A. (2016). miEAA: microRNA enrichment analysis and annotation. Nucleic Acids Res..

[22] Glazov E.A., McWilliam S., Barris W.C., Dalrymple B.P. (2008). Origin, Evolution, and Biological Role of miRNA Cluster in DLK-DIO3 Genomic Region in Placental Mammals. Mol. Boil. Evol..

[23] Winter J. (2015). MicroRNAs of the miR379-410 cluster: New players in embryonic neurogenesis and regulators of neuronal function. Neurogenesis.

[24] Kim J.Y., Park Y.-K., Lee K.-P., Lee S.-M., Kang T.-W., Kim H.-J., Dho S.H., Kim S.-Y., Kwon K.-S. (2014). Genome-wide profiling of the microRNA-mRNA regulatory network in skeletal muscle with aging. Aging.

[25] Mikovic J., Sadler K., Butchart L., Voisin S., Gerlinger-Romero F., Della Gatta P., Grounds M.D., Lamon S. (2018). MicroRNA and Long Non-coding RNA Regulation in Skeletal Muscle From Growth to Old Age Shows Striking Dysregulation of the Callipyge Locus. Front. Genet..

[26] Montag J., Hitt R., Opitz L., Schulz-Schaeffer W.J., Hunsmann G., Motzkus D. (2009). Upregulation of miRNA hsa-miR-342-3p in experimental and idiopathic prion disease. Molecular Neurodegeneration.

[27] Saba R., Goodman C.D., Huzarewich R.L.C.H., Robertson C.A., Booth S. (2008). A miRNA Signature of Prion Induced Neurodegeneration. PLoS ONE.

[28] Bellingham S.A., Coleman B.M., Hill A.F. (2012). Small RNA deep sequencing reveals a distinct miRNA signature released in exosomes from prion-infected neuronal cells. Nucleic Acids Res..

[29] Wang L.-L., Min L., Guo Q.-D., Zhang J.-X., Jiang H.-L., Shao S., Xing J.-G., Yin L.-L., Liu J.-H., Liu R. (2017). Profiling microRNA from Brain by Microarray in a Transgenic Mouse Model of Alzheimer’s Disease. BioMed Res. Int..

[30] Fu Y., Hu X., Zheng C., Sun G., Xu J., Liu R., Luo S., Cao P. (2019). Intrahippocampal miR-342-3p inhibition reduces beta-amyloid plaques and ameliorates learning and memory in Alzheimer’s disease. Metab Brain Dis..

[31] Tan L., Yu J., Tan M.-S., Liu Q.-Y., Wang H.-F., Zhang W., Jiang T., Tan L. (2014). Genome-Wide Serum microRNA Expression Profiling Identifies Serum Biomarkers for Alzheimer’s Disease. J. Alzheimer’s Dis..

[32] Cheng L., Doecke J.D., Sharples R., Villemagne V.L., Fowler C.J., Rembach A., Martins R.N., Rowe C.C., Macaulay S.L., Masters C.L. (2014). Prognostic serum miRNA biomarkers associated with Alzheimer’s disease shows concordance with neuropsychological and neuroimaging assessment. Mol. Psychiatry.

[33] Lugli G., Cohen A.M., Bennett D.A., Shah R.C., Fields C.J., Hernandez A.G., Smalheiser N.R. (2015). Plasma Exosomal miRNAs in Persons with and without Alzheimer Disease: Altered Expression and Prospects for Biomarkers. PLoS ONE.

[34] Liguori M., Nuzziello N., Introna A., Consiglio A., Licciulli F., D’Errico E., Scarafino A., Distaso E., Simone I.L. (2018). Dysregulation of MicroRNAs and Target Genes Networks in Peripheral Blood of Patients With Sporadic Amyotrophic Lateral Sclerosis. Front. Mol. Neurosci..

[35] Boese A.S., Saba R., Campbell K., Majer A., Medina S., Burton L., Booth T.F., Chong P., Westmacott G., Dutta S.M. (2016). MicroRNA abundance is altered in synaptoneurosomes during prion disease. Mol. Cell. Neurosci..

[36] Gao C., Wei J., Zhang B.-Y., Shi Q., Chen C., Wang J., Shi Q., Dong X.-P. (2016). MiRNA expression profiles in the brains of mice infected with scrapie agents 139A, ME7 and S15. Emerg. Microbes Infect..

[37] Lukiw W.J., Dua P., Pogue A.I., Eicken C., Hill J.M. (2011). Upregulation of micro RNA-146a (miRNA-146a), a marker for inflammatory neurodegeneration, in sporadic Creutzfeldt-Jakob disease (sCJD) and Gerstmann-Straussler-Scheinker (GSS) syndrome. J. Toxicol. Environ. Heal. Part. A.

[38] Saba R., Gushue S., Huzarewich R.L.C.H., Manguiat K., Medina S., Robertson C., Booth S. (2012). MicroRNA 146a (miR-146a) Is Over-Expressed during Prion Disease and Modulates the Innate Immune Response and the Microglial Activation State. PLoS ONE.

[39] Sierksma A., Lu A., Salta E., Eynden E.V., Callaerts-Vegh Z., D’hooge R., Blum D., Buée L., Fiers M., De Strooper B. (2018). Deregulation of neuronal miRNAs induced by amyloid-beta or TAU pathology. Mol. Neurodegener..

[40] Lukiw W.J., Alexandrov P.N. (2012). Regulation of complement factor H (CFH) by multiple miRNAs in Alzheimer’s disease (AD) brain. Mol. Neurobiol..

[41] Butovsky O., Jedrychowski M.P., Cialic R., Krasemann S., Murugaiyan G., Fanek Z., Greco D.J., Wu P.M., Doykan C.E., Kiner O. (2014). Targeting miR-155 restores abnormal microglia and attenuates disease in SOD1 mice. Ann. Neurol..

[42] Maffioletti E., Milanesi E., Ansari A., Zanetti O., Galluzzi S., Geroldi C., Gennarelli M., Bocchio-Chiavetto L. (2020). miR-146a Plasma Levels Are Not Altered in Alzheimer’s Disease but Correlate With Age and Illness Severity. Front. Aging Neurosci.

[43] Denk J., Boelmans K., Siegismund C.S., Lassner D., Arlt S., Jahn H. (2015). MicroRNA Profiling of CSF Reveals Potential Biomarkers to Detect Alzheimer’s Disease. PLoS ONE.

[44] Dong H., Li J., Huang L., Chen X., Li D., Wang T., Hu C., Xu J., Zhang C., Zen K. (2015). Serum MicroRNA Profiles Serve as Novel Biomarkers for the Diagnosis of Alzheimer’s Disease. Dis. Markers.

[45] Sanchez-Juan P., Bishop M.T., Kovacs G.G., Calero M., Aulchenko Y.S., Ladogana A., Boyd A., Lewis V., Ponto C., Calero O. (2015). A Genome Wide Association Study Links Glutamate Receptor Pathway to Sporadic Creutzfeldt-Jakob Disease Risk. PLoS ONE.

[46] Juźwik C.A., Drake S., Zhang Y., Paradis-Isler N., Sylvester A., Amar-Zifkin A., Douglas C., Morquette B., Moore C., Fournier A.E. (2019). microRNA dysregulation in neurodegenerative diseases: A systematic review. Prog. Neurobiol..

[47] Jia L.-H., Liu Y.-N. (2016). Downregulated serum miR-223 servers as biomarker in Alzheimer’s disease. Cell Biochem. Funct..

[48] Lusardi T.A., Phillips J.I., Wiedrick J.T., Harrington C.A., Lind B., Lapidus J.A., Quinn J.F., Saugstad J.A. (2016). MicroRNAs in Human Cerebrospinal Fluid as Biomarkers for Alzheimer’s Disease. J. Alzheimer’s Dis..

[49] Bauernfeind F., Rieger A., Schildberg F.A., Knolle P.A., Schmid-Burgk J.L., Hornung V. (2012). NLRP3 Inflammasome Activity Is Negatively Controlled by miR-223. J. Immunol..

[50] Duan Y., Kelley N., He Y. (2020). Role of the NLRP3 inflammasome in neurodegenerative diseases and therapeutic implications. Neural Regen. Res..

[51] Cheng C., Li W., Zhang Z., Yoshimura S., Hao Q., Zhang C., Wang Z. (2013). MicroRNA-144 Is Regulated by Activator Protein-1 (AP-1) and Decreases Expression of Alzheimer Disease-related A Disintegrin and Metalloprotease 10 (ADAM10)*. J. Boil. Chem..

[52] Hetz C., Russelakis-Carneiro M., Maundrell K., Castilla J., Soto C. (2003). Caspase-12 and endoplasmic reticulum stress mediate neurotoxicity of pathological prion protein. EMBO J..

[53] Schultz J., Schwarz A., Neidhold S., Burwinkel M., Riemer C., Simon D., Kopf M., Otto M., Baier M. (2004). Role of Interleukin-1 in Prion Disease-Associated Astrocyte Activation. Am. J. Pathol..

[54] Taylor D.R., Hooper N.M. (2006). The prion protein and lipid rafts (Review). Mol. Membr. Boil..

[55] Kovacs G.G., Gasque P., Ströbel T., Lindeck-Pozza E., Strohschneider M., Ironside J.W., Budka H., Guentchev M. (2004). Complement activation in human prion disease. Neurobiol. Dis..

[56] Mays C.E., Armijo E., Morales R., Kramm C., Flores A., Tiwari A., Bian J., Telling G.C., Pandita T.K., Hunt C.R. (2019). Prion disease is accelerated in mice lacking stress-induced heat shock protein 70 (HSP70). J. Boil. Chem..

[57] Llorens F., Ansoleaga B., Garcia-Esparcia P., Zafar S., Grau-Rivera O., López-González I., Blanco R., Carmona M., Yagüe J., Nos C. (2013). PrP mRNA and protein expression in brain and PrP(c) in CSF in Creutzfeldt-Jakob disease MM1 and VV2. Prion..

[58] Caughey B., Raymond G.J. (1993). Sulfated polyanion inhibition of scrapie-associated PrP accumulation in cultured cells. J. Virol..

[59] Lehmann S. (1995). Sulfated Glycans Stimulate Endocytosis of the Cellular Isoform of the Prion Protein, PrP^C., in Cultured Cells. J. Boil. Chem..

[60] Filali H., Martín-Burriel I., Harders F., Varona L., Lyahyai J., Zaragoza P., Pumarola M., Badiola J.J., Bossers A., Bolea R. (2011). Gene Expression Profiling and Association with Prion-Related Lesions in the Medulla Oblongata of Symptomatic Natural Scrapie Animals. PLoS ONE.

[61] López-Pérez Ó, Bernal-Martín M., Hernaiz A., Llorens F., Betancor M., Otero A., Toivonen J.M., Zaragoza P., Zerr I., Badiola J.J. (2020). BAMBI and CHGA in Prion Diseases: Neuropathological Assessment and Potential Role as Disease Biomarkers. Biomol..

[62] Almeida L.M., Basu U., Khaniya B., Taniguchi M., Williams J.L., Moore S., Guan L.L. (2011). Gene Expression in the Medulla Following Oral Infection of Cattle with Bovine Spongiform Encephalopathy. J. Toxicol. Environ. Heal. Part. A.

[63] Marbiah M.M., Harvey A., West B.T., Louzolo A., Banerjee P., Alden J., Grigoriadis A., Hummerich H., Kan H.-M., Cai Y. (2014). Identification of a gene regulatory network associated with prion replication. EMBO J..

[64] Théry C., Witwer K.W., Aikawa E., Alcaraz M.J., Anderson J.D., Andriantsitohaina R., Antoniou A., Arab T., Archer F., Atkin-Smith G.K. (2018). Minimal information for studies of extracellular vesicles 2018 (MISEV2018): A position statement of the International Society for Extracellular Vesicles and update of the MISEV2014 guidelines. J. Extracell. Vesicles.

[65] Liu S., Hossinger A., Göbbels S., Vorberg I. (2017). Prions on the run: How extracellular vesicles serve as delivery vehicles for self-templating protein aggregates. Prion.

[66] Beraldo F.H., Arantes C.P., Santos T.G., Machado C.F., Roffe M., Hajj G.N., Lee K.S., Magalhaes A.C., Caetano F.A., Mancini G.L. (2011). Metabotropic glutamate receptors transduce signals for neurite outgrowth after binding of the prion protein to laminin gamma1 chain. FASEB J..

[67] Um J.W., Kaufman A.C., Kostylev M., Heiss J.K., Stagi M., Takahashi H., Kerrisk M.E., Vortmeyer A., Wisniewski T., Koleske A.J. (2013). Metabotropic glutamate receptor 5 is a coreceptor for Alzheimer abeta oligomer bound to cellular prion protein. Neuron..

[68] Lee W.S., Bae Y.C., Suk K., Lee W.-H. (2019). Axon Guidance Molecules Guiding Neuroinflammation. Exp. Neurobiol..

[69] Kim T.-K., Lee I., Cho J.-H., Canine B., Keller A., Price N.D., Hwang D., Carlson G.A., Hood L. (2020). Core transcriptional regulatory circuits in prion diseases. Mol. Brain.

[70] Tian C., Liu D., Chen C., Xu Y., Gong H.-S., Chen C., Shi Q., Zhang B.-Y., Han J., Dong X. (2013). Global transcriptional profiling of the postmortem brain of a patient with G114V genetic Creutzfeldt-Jakob disease. Int. J. Mol. Med..

[71] Lopez-Bendito G., Flames N., Ma L., Fouquet C., Di Meglio T., Chédotal A., Tessier-Lavigne M., Marin O. (2007). Robo1 and Robo2 Cooperate to Control the Guidance of Major Axonal Tracts in the Mammalian Forebrain. J. Neurosci..

