# Supplementary material for: MicroRNA Alterations in a Tg501 Mouse Model of Prion Disease
Source: Biomolecules. 2020 Jun 15;10(6):908. doi: 10.3390/biom10060908 (PMC7355645; doi:10.3390/biom10060908)
Supplement: Supplementary file 1 [file biomolecules-10-00908-s001.pdf]

Supplementary Information

Table 1. Comparison of miRNA detection carried by the two platforms.

Platform	Total Reads	miRNAs detected	% of miRBase[1] annotated	% of miRBase HC[2]
Chimira[3]	1.5×10^8	1755	91.6	95.4
Oasis 2.0[4]	1.45×10^8	1702	89.2	94.6

[1]miRBase 21[12],[2]High confidence miRNAs [3]Chimira [15], [4]Oasis 2.0 [13,14].

Table 2. List of miRNAs verified by qRT-PCR and corresponding TaqMan Assays used in the study.

miRBase Accession	miRNA	miRNA Sequence	TaqMan Assay #
MIMAT0000158	miR-146a-5p	UGAGAACUGAAUUCCAUGGGUU	000468
MIMAT0000590	miR-342-3p	UCUCACACAGAAAUCGCACCCGU	002260
MIMAT0003734	miR-667-3p	UGACACCUGCCACCCAGCCCAAG	001949
MIMAT0000665	miR-223-3p	UGUCAGUUUGUCAAAUACCCCA	002295
MIMAT0000215	miR-186-5p[1]	CAAAGAAUUCUCCUUUUGGGCU	002285
MIMAT0003185	miR-369-5p[1]	AGAUCGACCGUGUUAUAUUCGC	001021
MIMAT0000069	miR-16-5p[2]	UAGCAGCACGUAAAUAUUGGCG	000391
MIMAT0000010	cel-miR-39-3p[2]	UCACCGGGUGUAAAUCAGCUUG	000200

[1] Normalizer for CNS tissues, [2]Spike-in normalizer for plasma.

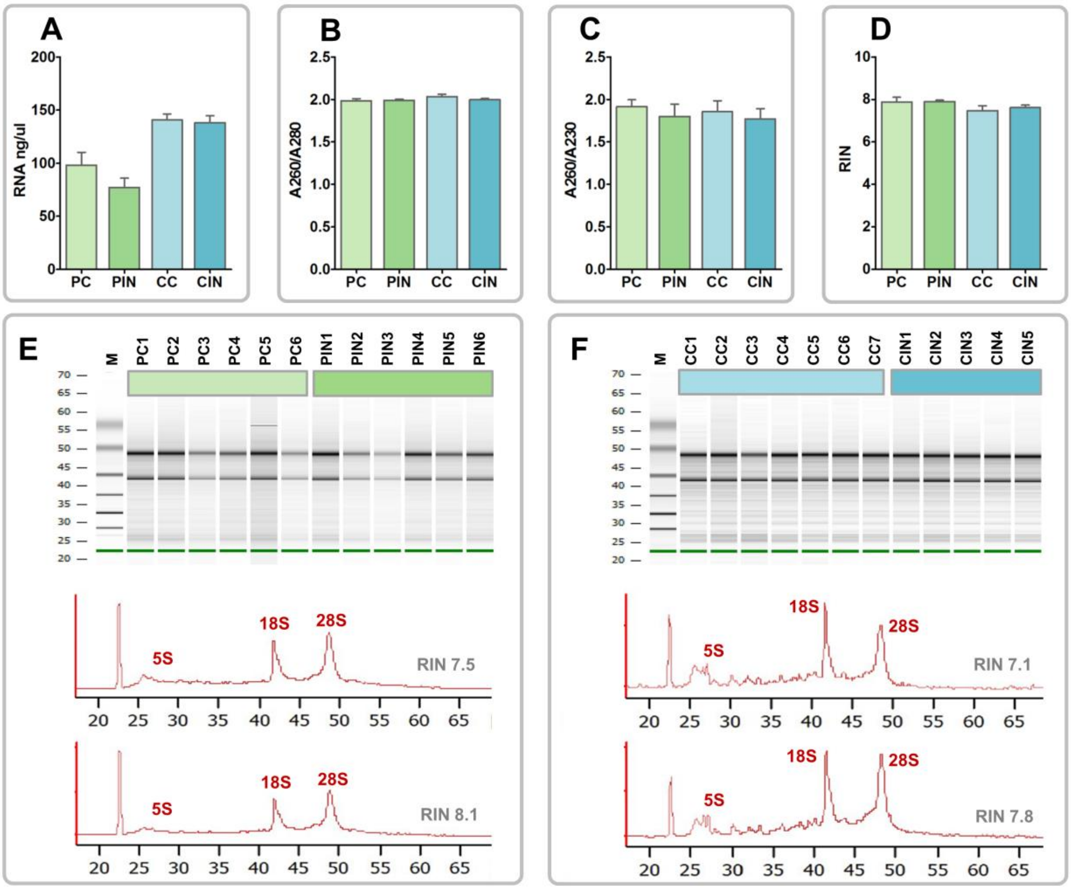

Figure S1. Quality control of cervical spinal cord RNA samples from Tg501 mice. The preclinical samples are indicated in shades of green (pale green, control (PC); dark green, inoculated (PIN)) and symptomatic/clinical samples in shades of blue (pale blue, control (CC); dark green, inoculated (CIN)). (**a**) Mean RNA quantity in nanograms per microliter; (**b**) Absorbance ratio A260/A280 and (**c**) absorbance ratio A260/A230 for each sample group; (**d**) Mean RNA integrity value (RIN) for each sample group. The error bars in (**a**)–(**d**) refer to standard error of mean. (**e**) Electrophoresis file from preclinical samples and graphs representing the lowest and the highest RIN values (range 7.5–8.1); (**f**) Electrophoresis file from clinical samples and graphs representing lowest and highest RIN values (range 7.1–7.8). In (**e**)–(**f**), the position of the 22 bp bands excised for sequencing in green and the peaks corresponding to the main rRNA areas are indicated in red.

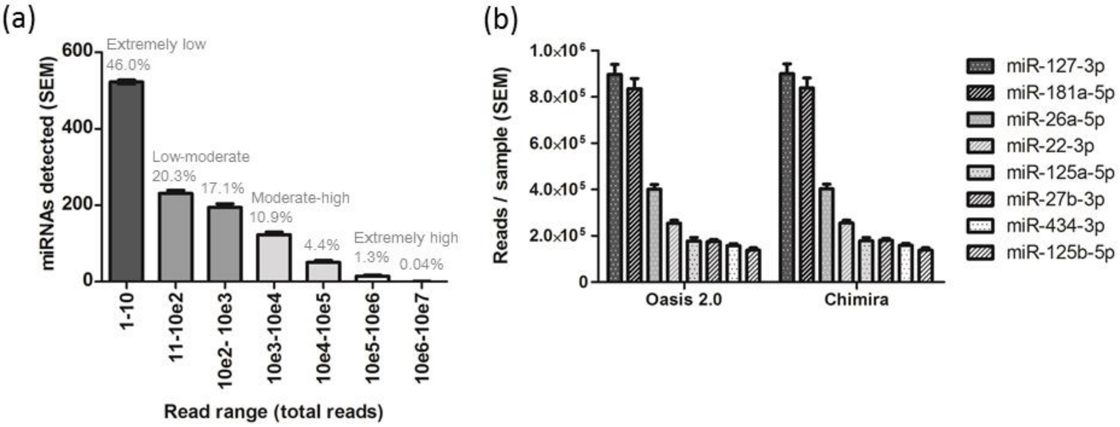

Figure S2. Read count range and most abundant miRNAs in Tg501 cervical spinal cord samples. **(a)** Read count range of detected miRNAs. The bars represent the mean number +/- standard error of mean of detected number of miRNAs across the all experimental mice used in the study and separated in the categories based on read count range. Approximately 46% of the detected mature miRNAs were found to be present in extremely low levels (1-10 reads per sample), ~37% were expressed in low to moderate levels ($11–1 \times 10^3$ reads), ~15% in high levels ($1 \times 10^3–10^5$ reads) and ~1.3% in extremely high levels ($1 \times 10^5–10^7$ reads). **(b)** Top 8 miRNAs based on their expression levels as detected by Oasis 2.0 and Chimira. For each miRNA, the bars represent the mean number of reads per mouse +/- standard error of mean across the all experimental mice used in the study. Both platforms detected the same set of highly expressed miRNAs whose expression accounts for 49% of the total experimental reads.

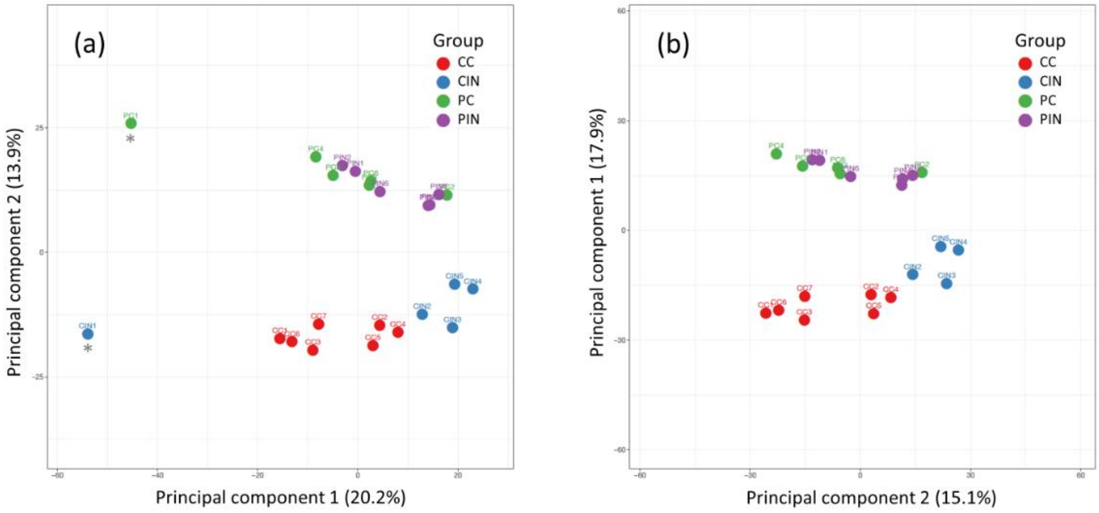

Figure S3. Principal component analysis of read counts from cervical spinal cord of Tg501 mice. **(a)** The experimental raw RNA sequencing reads analysis revealed that two samples (PC1 and CIN1, grey asterisks) were outliers and these samples were excluded from downstream data analysis. **(b)** The data re-analyzed after removal of outlier samples. The result is shown for Clustvis-produced raw counts and the same result was obtained from counts produced by Oasis 2.0 (data not shown). Abbreviations: CC, clinical control; CIN, clinical inoculated; PC, preclinical control; PIN, preclinical inoculated.
